# Do critical incidents lead to critical reflection among medical students?

**DOI:** 10.1080/21642850.2021.1899827

**Published:** 2021-03-18

**Authors:** Anthony Montgomery, Karolina Doulougeri, Efharis Panagopoulou

**Affiliations:** a School of Social Sciences, Humanities and Arts, Department of Educational & Social Policy, University of Macedonia, Thessaloniki, Greece; b Eindhoven School of Education, Eindhoven University of Technology, Eindhoven, Netherlands; c Aristotle University of Thessaloniki, Faculty of Health Sciences, Aristotle University of Thessaloniki, School of Medicine, Thessaloniki, Greece

**Keywords:** Critical reflection, professional identity, doctor–patients interactions, medical education

## Abstract

**Context:**

Medical students are exposed during their training to a wide range of experiences and behaviors that can affect their learning regarding professionalism and their behavior and attitudes towards patient-centered care. The aim of the study is to explore learning associated with critical incidents and levels of critical reflection among medical students.

**Approach:**

Medical students’ were invited to narrate a critical incident and reflect on the learning associated with it. All students’ narratives were audio-recorded and analyzed thematically. Mezirow’s theory of transformative learning was used to analyze the level of reflection reached in students’ narratives.

**Findings:**

For the present analysis critical incidents narrated by 70 clinical students (4th–6th year) were included. Fifty-two of them were females. Students’ experiences are derived from three types of interactions: observed interactions between doctors and patients, personal interactions between students and patients, and interactions between doctors and students. Reflections deriving from the experiences included: behaving to patients as humans not as cases, emotional aspects of care, doctors as role models, skills needed when working under pressure, ‘tasting’ the real profession, emotional management, the importance of communication skills, teaching qualities of doctors, becoming a doctor, and reflections of future practice. Analyzing the actual level of reflection indicated that only 32 (45.7%) students were categorized as reflectors.

**Conclusions:**

Student interactions with doctors and patients provide insights about; the daily experience of being a doctor, the most common challenges, what qualities are necessary for being a doctor and what do they need to develop their professional identity. However, it is noteworthy that while the majority of students shared a critical incident crucial to their professional development, there is little evidence of critical reflection.

## Introduction

Reflecting critically about one’s own practice is the starting point for gaining new perspectives in the daily routines of working professionals (Dewey, 1993). Critical reflection is a skill that is typically taught/used in the humanities and social sciences, and it is one that has been identified as a crucial factor in leadership development (Li, Gray, Lockwood, & Buhalis, 2013), management and entrepreneurial learning (Franz, 2010), and learning within teams (Schippers, Den Hartog, & Koopman, 2007). However, it is a skill that can have a significant benefit for medical education. The delivery of medicine is becoming increasingly interdisciplinary, demands high levels of team working and should involve patients as much as possible. Critical reflection has the potential to enable medical students to more easily find the connections between clinical practice and quality of care, and maximize the learning that arises from near misses and medical mistakes.

Medical students are exposed, during their training, to a variety of conflicting experiences that can have a profound impact on their professional development.

In particular, research suggests that students typically experience conflicting messages about professional behavior within in-patient hospital settings (Hafferty, 1998). Professionalism dilemmas reported in the literature involve students experiencing conflict between pre-clinical teaching and actual patient care, students witnessing or participating in breaches of patient dignity or patient safety, and students experiencing abuse.

Conflicting messages affect students at different levels. At a behavioral level studies have shown that students often respond with inaction for fear of jeopardizing their grades and career prospects (Doulougeri, Panagopoulou, & Montgomery, 2016). At an emotional level, research also shows that medical students feel anger, resentment, and guilt in the face of professional dilemmas (Rees, Monrouxe, & McDonald, 2013). This in turn leads to moral distress, which in turn manifests as negative emotional reactions arising from the thwarting of an individual’s desire to take the right course of moral action (Gaufberg, Batalden, Sands, & Bell, 2010; Monrouxe, Rees, & Hu, 2011; Wiggleton et al., 2010). However, very little is known about the way these conflicting experiences are processed at a cognitive level. In other words, to what extent do medical students reflect or learn from the conflicting experiences they encounter during their education?

Mezirow (1991) defines reflection as ‘the process of critically assessing the content, process, or premise(s) of our efforts to interpret and give meaning to an experience’ (p. 104). Mezirow’s model views reflection as an essential part of the learning process. The model identifies different levels of reflection and sorts them into a taxonomy in which learning behavior is viewed as a transformative process. In this bottom-up model, there are three non-reflective levels (habitual action, thoughtful action, and introspection) and three reflective levels (content reflection, process reflection, and premise reflection). According to Mezirow, ‘content reflection’ is an examination of the content or description of a problem. ‘Process reflection’ involves checking on the problem-solving strategies that are being used. ‘Premise reflection’ leads the learner to a transformation of meaning perspectives. Mezirow’s model describes assessing levels of reflection in 3 distinct categories: non-reflector, reflector, and critical reflector. Within healthcare, the model has been used to measure reflection primarily with journals and blogs used by nursing students (Jensen & Joy, 2005; Wong, Kember, Chung, & Yan, 1995).

Previous studies on conflicting experiences in medical education have used various methods such as questionnaires, focus groups and student essays on ethics (Rees et al., 2013; Gaufberg et al., 2010; Monrouxe et al., 2011; Karnieli-Miller et al., 2011; Karnieli-Miller, Vu, Holtman, Clyman, & Inui, 2010). A methodology, which can provide in-depth information on the impact of conflicting experiences, is critical incidents analysis. Critical incident reports are short narrative accounts used in medical education. They represent short narratives of incidents that medical students judge to be important learning experiences. They present us with a ‘window’ into the formal and informal messages transmitted via healthcare. Critical and/or memorable incidents represent the point at which an organization reveals important information as to what attitudes and behaviors are valorized (Brady, Corbie-Smith, Branch, & William, 2002). Mezirow posited that critical incidents enhance learning by providing access to experiences that facilitate personal growth. He called this transformative learning.

Within the above framework, the present study aims at using critical incident analysis to firstly explore the types of learning associated with conflicting experiences, and secondly to identify the levels of critical reflection achieved by medical students.

## Method

### Sample and procedure

The present study is part of a larger project exploring memorable experiences, emotions, and learning in both clinical and pre-clinical students. The entire medical student body from a Medical school in Northern Greece was eligible to participate. Medical students were invited to participate via announcements in the university online platforms. Interested students contacted the research team to arrange an appointment for the interview.

### Training the interviewers – peer interviewing

Our previous experience with qualitative studies with medical students suggested that medical students might be reluctant to share a critical or memorable incident to someone outside of the medical school or someone involved in the formal medical curriculum, especially if the incident was regarded as negative. For this reason, we decided that engaging other medical students to act as interviewers would be less confrontational for participants, enabling them to share possible disturbing emotions and experiences (Byrne, Brugha, Clarke, Lavelle, & McGarvey, 2015).

Medical students participating in an elective course of Communication skills were invited to act as interviewers. The course is an elective for both pre-clinical and clinical students. Participation in the study as interviewer was voluntary and it was not associated with any incentive. EP, an experienced researcher and professor in communication skills, provided the interviewers with a training workshop (1 h 30 min) including theoretical and practical elements of qualitative interviewing for research purposes, where the students practiced with each other, received feedback from their peers and then reflected on the process. In this workshop they were introduced to the principles of qualitative interviewing as well as ethics in research and they were provided with relevant reading material.

In total 50 (from a class of 93) students volunteered to be interviewers and received the training. Among the interviewers 35 were female students and 15 were male students; 13 were pre-clinical students and 37 were clinical students. Taking into account the number of participants included in the study every interviewer conducted approximately two interviews. The interviewers were provided with an interview guide for assessing critical incidents, emotions and learning in medical students (see Table 1). The assignment of interviewers and students was quasi-random and the availability of time of both parties was a factor in matching the interviewers and students.

**Table 1.** Interview guide for assessing critical incidents, emotions and learning in medical students.

General Instructions for peer interviewers: be sure to leave adequate time for the interview check audio recording apparatus and test it welcome the participants explain the purpose of the study and the process of the interview explain confidentiality obtain consent before recording follow the interview guide conclude the interview with care and respect if any issues come up, refer the participant to the study coordinators		
	Questions	Notes for interviewers
1	Describe a memorable incident that occurred during your studies	[ ]In case participant asks, the incident can be positive or negative and it could take place at any stage of their studies [ ]Clarify when the incident took place [ ] Clarify where the incident took place [ ] Clarify who was involved
2	After encountering this incident, what was your reaction? What was the reaction of others?	[ ] Explore feelings associated with the incidents [ ]Explore the initial reaction of participants [first thoughts, feelings, behaviors]
3	Did you do anything/did others do anything?	[ ] Explore how the participant or other people involved in the incident behaved as a response to it
4	Looking back on the event, what are your thoughts and emotions about it	[ ] If unanswered in previous questions, give a summary of the event and explore emotions and thought associated with it. [ ]Explore what is the current impression of the participants regarding the incident

Note: Interview guide for peer interviewers. Description of data: an interview guide was developed to facilitate the peer interviewers in conducting the interviews. The guide included general instructions for the interview process, the core questions of the interview, as well as a checklist of important information that peer interviewers should collect at each stage of the interview.

### Data collection

According to the Aristotle University Research Ethics Board, ethical approval for medical education research, involving medical students, where no intervention is applied, is not required. When interested students contacted the research team, the purpose of the study was explained to them in detail, anonymity and confidentiality were guaranteed. Participation in the study was voluntary and no incentive was given for participation.

During the interview participants were asked to describe a critical/memorable that occurred during their studies, without suggesting whether it had to be a positive or negative event. In addition, medical students were invited to reflect on this event and report what had they learnt out of it. Interviewers assigned a special code to each audio file, containing the interview, and gave it to the first and second author without any personal identification. In total 104 students consented to be audio recorded.

### Data analysis

The focus of the present paper was on medical students’ conflicting experiences within clinical settings, so for the present analysis only incidents reported by clinical students, were included in the analysis. This resulted in 70 critical incidents narrated by 70 students at clinical stage of medical education.

The second author, who does not work in the medical school and has sole access to the audio recordings, transcribed verbatim all interviews. Two researchers (KD and EP) independently analyzed and coded the data and compared their results. The authors independently undertook an inductive thematic analysis of the data, reading, re-reading, coding, and categorizing the data (Braun & Clarke, 2006). The independently generated themes were reviewed by the three authors and final agreement was reached. Interrater reliability was high reaching 85%. Disagreements were resolved by the input of an independent researcher. Saturation of themes emerged after the first 20 experiences. Given that the focus of this analysis was conflicting experiences within clinical settings, only memorable incidents reported by clinical students, describing such an experience were included in the analysis. The included transcripts were subsequently categorized into three categories: Observed interactions between doctor and patient, personal interactions between student and patient, and interactions between doctors/trainers and students.

### Levels of reflection and learning

During the first level of analysis every interview was examined for evidence of utilization of one or more of the elements of reflection as defined by Mezirow’s model. The next level of analysis involved placing students into one of three broader categories of non-reflectors, reflectors and critical reflectors as defined by Mezirow (1991) and utilized by Wong et al. (1995). Using the taxonomy of Mezirow’s, six levels can be identified. The first 3 levels are considered non-reflective levels (habitual action, thoughtful action, and introspection). Students whose narratives were categorized in one of these levels were considered non- reflectors. Students exhibiting content and process reflection in their narratives were categorized as reflectors and finally students who were able to engage in critical reflection were categorized as critical reflectors.

## Ethical statement

According to the Aristotle University Research Ethics Board, ethical approval for medical education research, involving medical students, where no intervention is applied, is not required.

## Results

### Demographic characteristics of participants

For the present study 70 critical incidents, narrated by 70 medical students in clinical years were included in the analysis. All the narratives included conflicting experiences within clinical settings. From the participating students 18 were male and 52 were female. 45 students were in the 4th year of medical school, 16 students in the 5th year and 9 in the 6th year of medical school.

### Themes of learning

**Figure 1.**
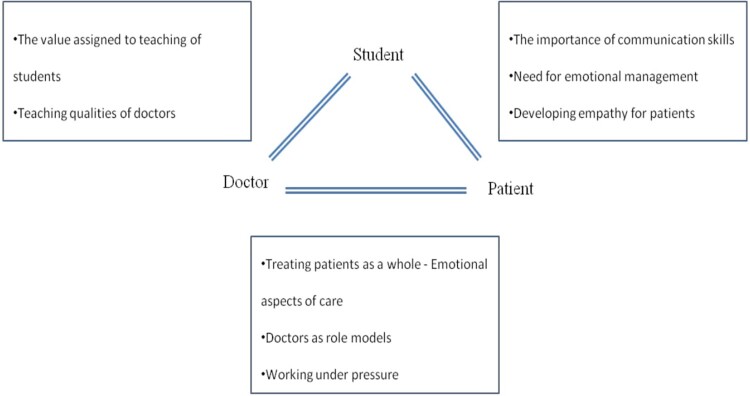
Theme emerged within each type interaction.

Figure 1 summarizes the themes emerged within each type of interaction. Table 2 summarizes all learning themes present in the critical incident narratives. In the following sections the learning associated with different types of interactions within hospital settings.

**Table 2.** Levels of reflection.

		Frequency	Percent
Non-reflectors	Thoughtful action Introspection Content reflection Process reflection Premise reflection Total	19	27.1
		19	27.1
Reflectors		18	25.7
		13	18.6
Critical Reflectors		1	1.4
		70	100.0

### Lessons learnt from observed interactions between doctors and patients

The main learning themes associated with interactions among doctors and patients included (a) Treating patients as whole- Emotional aspects of care (b) Doctors as role models and (c) Working under pressure.

Both positive and negatives narratives prompted students to respect patients’ needs, preferences:

Doctors should exhibit respect towards patients and not treat them only as medical cases. They should treat them as humans.

They also prompted them to reflect that part of doctor’s job is to provide reassurance and address the emotional needs of a patient.

I realized that among a doctor’s duty is not only to heal the patient but also be present and support him emotionally if needed.

I will always remember the behavior of the doctor and I would like in the future to spend more time with my patients in order to reassure them.

Through their narratives medical students seem to elaborate on the qualities of a ‘good doctor’. Those included being a good listener, having patience, showing respect, and taking time to address all patients’ concerns.

A good doctor should be patient, a good listener and understanding, not rude but always patient and ready to listen to what the patient wants to share with him.

In addition, a main theme concerning the qualities of a good doctor was the ability to manage successfully his emotions and perform under pressure.

Many times during our career we will face situations that are emotionally intense and we have to find the way to handle our emotions in order to do our job.

### Lessons learnt from personal interactions between students and patients

From these incidents students learnt that the importance of communication skills and emotional management for effective patient care.

Students realized that interactions with patients require not only clinical knowledge and skills but also the ability to communicate effectively and reassure the patient.

The patient did not want me to perform the venupucture. I knew I could do it, but I did not know how to convince him and defend myself. I learnt that it does not matter how confident I feel for my skills I have also to use the appropriate arguments to convince the patient who, understandably, is worried.

Students reported several conflicting emotions associated with the first contact with students, such as nervousness, excitement, and sadness. In addition, feeling insecure about their ability to handle the situation made students realize the importance of managing their emotions.

What I learnt from this event is that your life can change in a moment. Apart from that, I couldn’t sleep the whole night, I was thinking of the patient for many days. I realized that I shouldn’t identify that much with my patient, I should be tougher. If I was tougher it would be better for me because I could sleep in the nights but also for her because I would be more available for her.

However, there were students who regarded dissociation of feelings as an unavoidable side effect of care giving.

Unfortunately what I learnt was that all of us, or some of us, will be forced to disengage from our feelings and do the job that we are paid for and not our job in its essence.

Direct contact with patients helped students to adopt the patients’ perspective and develop empathy.

For the first time I realized how insecure a patient might feel, this incident helped me see also the perspective of the patient; I wouldn’t like to be sick and being treated like this.

When I will become a doctor I would like to give more attention to my patients, to show more interest for them because I would not like to be in their situation, coming back home and being stressed for my health and not having anyone to address my questions and trust.

### Lessons learnt from personal interactions between students and doctors

In this category of incidents medical students mainly reflected on the difference they observe in doctors depending whether they are engaged in clinical or teaching activities, and the teaching qualities that medical educators should exhibit.

Several times medical students described incidents where they felt a burden for their teachers. Many of them reported that teaching for many doctors was not considered as important as treating their patients and that reflected on the quality of teaching and the motivation of students. When encountered with incidents of haphazard teaching students felt that clinical teachers did not respect them enough. Many of the students stated that they feel abandoned without guidance. Students expressed their need to feel their teachers are approachable and interested in them.

What I learnt is that teachers should be approachable and it is their duty to answer to our questions and respect us. It will be helpful if they could empathize and reflect how they were feeling in our age, because if a student is intimidated and is hesitating to ask questions, he is not going to learn.

Positive role models with regard to teaching were medical teachers showing engagement, motivation, and willingness to spend time with students. Doctors who were clinically competent but also empathetic and respectful were valued positively.

I was really impressed by a teacher who was a bit late in our class, he was the only one who apologized and he stayed longer after the end of the class to answer questions, repeating how sorry he was for his delay. I realized that even though I am disappointed by our teachers, there are some, who after a night shift or a long day still have the motivation to teach us.

### Critical incidents reported by students: level of reflection in students’ narratives

Firstly, the content of medical students’ narratives was analyzed to identify the level of reflection. Medical students were categorized into non-reflectors and reflectors. Below we provide specific examples of the different types of reflectors and non-reflectors we identified.

Mezirow’s taxonomy labeled students as: non-reflectors (no evidence of reflective thinking), reflectors (evidence of relating experience to learning opportunities) and critical reflectors (evidence of integrating reflective outcomes in professional behavior).

Table 2 provides details about the frequencies and percentages of critical incidents categorized across the different levels of reflection. As shown in Table 2, 54.3% of the students were categorized as non-reflectors according to Mezirow’s taxonomy. Table 3 provides examples of the themes and reflections that emerged from medical students’ narratives.

**Table 3.** Themes and reflection emerged from medical students’ narratives.

Themes	Short excerpts from students narratives
Observed interactions between doctors and patients	
Doctors not answering questions or addressing concerns of patients	*The doctor announced to a patient without -approaching her- that she had to move to the surgery department. When the patient, worried ask for some explanations, he started shouting ‘I said a very simple phrase, what didn’t you understand? … I don’t care where you will go, I just want you to leave this room*
Doctors concealing diagnosis or prognosis	*I realized that the patient was not aware of the prognosis of her disease. When I asked the doctor, ‘why don’t you inform the patient for her condition?’ the answer was: ‘This is the policy we follow in this clinic’.*
Doctors behaving to patients in a rude, disrespectful way	*The supervisor made a derogatory comment for the educational level and ethnicity of the patient in front of him*
Doctor minimizing patient’s pain or ignoring patients in pain from not being an urgent situation	*The patient was suffering from pain and when I asked the doctor why don’t we provide him some with some painkillers he said ‘he is exaggerating, he just feels a small bite that’ s all’*
Doctors making racist attributions for patient	*The doctor said that because of his ethnic background, the patient should undergo more exams, because people in his ethnicity usually lie for their health*
Doctors making fun of patient characteristics behind patients’ back	*Doctors constantly making fun of patients about their clothes or their hair. They always comment on whatever characteristic the patient might have.*
Doctors submitting patients into repeated exams for meeting educational needs	*The patient seemed exhausted and almost ready to cry after all this repeated exams. When he asked the doctor to stop, the doctor answered ‘The students have also to learn’*
Doctors ignoring patients complaint or reluctance to undergo repeated procedure for educational needs	*The patient was not being admitted to surgery because it was a good learning experience for us, even though we could see that he was suffering.*
Doctors exhibiting reassuring and supporting patients in distress	*The patient looked really worried and she asked the doctor ‘Doctor, am I going to die? So the doctor approached her, held her hand and said: Don’t be afraid, we are doing whatever is possible to help you*
Doctors addressing patients’ concerns in a clear and understandable way	*The patient had visited the doctor to show him the results of her exams. The patient was really worrying and the doctor was trying to reassure her that everything in her exams was normal. To convince her, he devoted enough time and he even brought some books from his bookcase and showed her pictures of abnormal exams and invited her to compare together the differences. She immediately seemed more relieved and I found this idea of the doctor brilliant.*
Doctors reacting fast and accurately in urgent situations	*A man was brought in the emergency department with a heart arrest. Doctors without losing time, did whatever was possible to save him. Finally the patient opened her eyes and she thanked the doctor for saving her life. I felt so grateful that I was there.*
Doctor freeze and being unable to react	*They brought us this girl after a serious car accident. The parents were with her crying and shouting. The resident on shift was just standing there unable to react. She was not answering to the questions of the others around her. I was really worried. I was thinking: is she going to collapse now?*
**Personal interactions between students and patients**	
Contact with a person in terminal disease for the first time	*It was the first time, I was seeing someone in such a terminal situation. I felt so sad for how short life can be for some people*
Contact with a patient in pain	*The patients was moaning from pain, it was really penetrating*
Contact with a patient dying	*The patient had a blue green color, his wife was shouting and I could not believe I was seeing a person dying in front of me.*
**Challenges in communication with patients**	
Taking history from a patient in severe situation or an older patient	*The patient was quite old and tired and was not very collaborative in answering my questions or undergoing physical examination. At the end, I quitted from trying*
Contact with a difficult patient	*The patient was very weak but when she asked how old I was and I told her I am still a medical students, she started pushing my hand away not allowing to do blood taking*
Contact with a patient with language barriers	*The patient did not speak xxx or English, so we tried to replace the verbal interaction with some non-verbal cues during examination*
**Personal interactions between students and medical educators**	
Medical educators not arriving in lesson, or arriving late	*I feel that no one in Medical School respects the effort we do to enter and succeed in this faculty. they simply do not care about us*
Medical educators arriving unprepared for the lesson	
Medical educators seemed bored and unmotivated while teaching	*Medical teachers should realize their responsibility as educators and show more willingness to devote some time in us. If you do not run after them … no one wants to teach you anything*
Medical educators making sexist comments regarding specialty choice	*During a conversation we had with a teacher of us, he told us that according to him, women should not follow surgical specialties. I thought ‘Oh God, who allows to those people to teach future doctors?’*
Medical educators embarrassing students during bedside teaching for not answering questions	*I had a question so I asked the professor. Then she started saying that she will not accept such stupid questions anymore and that what I asked was very silly. I did not react then to defend myself and after that, every time I have a question I hesitate to ask and I am wondering whether my question is silly or whether I was supposed to know the answer already.*

### Non-reflectors

In our sample, students categorized as non-reflectors provided narratives corresponding to thoughtful action and introspection categories. We did not have any example of habitual action. Habitual action includes a description of an act performed without thought or having to focus or a description of the course of events. Given that in our study we asked students to describe a critical event and reflect on it, we did not have an example of habitual action.

#### Thoughtful action

This category describes narratives of students who after observing a critical incident they made a statement drawing up existing theoretical knowledge without consciously processing alternative options or justifying why a certain choice should be made. No interpretation of their rationale is provided. For example, after observing a rude behavior exhibited by a doctor to a patient, a reflection such as ‘doctors should treat patient with more respect’, is logical and expected and draws upon theoretical knowledge of patient-centered care but it does not explain why it is important for a doctor to exhibit respect to a patient.

#### Introspection

This category entailed narratives of students that referred to students’ thoughts about oneself, one’s own thoughts or feelings about performing a task or observing a critical incident. In these narratives, no comparison between the critical incident and/or one’s previous experiences, nor are there any thoughts as to why these feelings occur or what they might lead to. An example of this is the following:

It was the first time I was seeing someone in such a terminal situation. I felt so sad for how short life can be for some people.

This statement connects the actual experience with student’s feelings without providing any elaboration on what these feelings actually meant to him or whether these feelings were associated with any subsequent action or behavior.

### Reflectors

#### Content reflection

This category included students who explained what they thought, felt or acted when facing a critical incident. They questioned or interpreted their behavior and they consciously chose a certain course of action.

I approached an instructor to ask some details for the class and some questions about the format of the upcoming exam. He did not seem willing to devote time to me. He was quite rude and I felt very frustrated. However, I thought that I would not gain anything from reacting also badly so I asked again politely, hoping that I will get an answer.

The student facing a rude behavior by an instructor felt frustrated. Despite her negative feelings she consciously chose to continue insisting for answers politely knowing that arguing with the instructor would not help her.

#### Process reflection

In this category students described a critical incident and they elaborated on their thoughts, feelings, actions and they evaluated them with regard to their effectiveness. For example, a student who had to deal with a disruptive family member described it as follows:

Once I had to take history from a patient whose sister was constantly interrupting him to add more details to his answers. Initially I felt irritated but I thought that would not help me continue the process so I did not react and I let her continue. The reason was that I realized that in certain moments her assistance was useful because she was adding valuable information but there were moments that she was confusing the patient further. So, I learnt that the accompanying person can be useful as long as you can deal with the patient and not be distracted from your work. It is important to set some limits.

In this example, the student acknowledged that being interrupted constantly by the accompanying person was irritating. She weighted the pros and cons of having the accompanying person present to the process and decided to allow her being there given that she could add valuable information. On the other hand, the student recognized the importance to of setting boundaries in order to achieve a good outcome.

### Critical reflectors

#### Premise reflection

This category included only one narrative. To be categorized as critical reflection the student provided a detailed analysis of the problem as well as consequences associated with the problem and alternative choices. In a critical reflection the person reinterprets the current situation in a way that a different course of action would be followed next time.

Once my supervisor asked me to provide stitches for a patient who had been just admitted to the hospital. When the patient saw me, he reacted really badly and he refused to allow me to do the stitches, calling me a kid. I initially got shocked; however, I tried to convince him. I realized that the patient was scared and probably did not have anything against me, but it was normal to question my skills due to my young age and the fact that I was only a medical student. I tried to convince him by saying that I have performed this procedure before and that I was there to help him. I tried to appear confident. I realized that several patients might question my skills. From this experience I learnt that by using the appropriate arguments and clinical experience you can convince even a negative patient but it is important to exhibit patience and confidence to yourself.

## Discussion

Using the methodology of critical incidents reporting, the present study explored the levels of reflection, and perceived learning associated with conflicting experiences during medical education.

As supported by previous studies, the majority of incidents reported by students were related to breaches of patient-centered care and were part of the hidden curriculum of medical education (Rees et al., 2013; Gaufberg et al., 2010; Karnieli-Miller et al., 2011). Medical education requires discourses of professionalism which illuminate the impact of the hidden curriculum, to enable students to identify and resist its negative impacts. Viewing the learning experience of students can help clinicians appreciate how their attitudes play an important role in influencing physician behavior.

Participants also discussed learning associated with their interactions, – either direct ones between doctors and patients, or observed ones between doctors and patients. This is in agreement with research on ‘communities of practice’ which approaches the concept of learning as a sociocultural experience (Lave & Wenger, 1991).

Memorable lessons associated with the above incidents, included the importance of a holistic approach to patient care, communication skills and the successful management of patient emotions. Being able to regulate their own emotions, and work under pressure, were lessons related to their qualities as future doctors.

### Limitations

The study was conducted in one medical school so results are limited to the learning environment of this medical school. We cannot exclude a retrospective bias given that we asked students to recall an event and given that the sample was self-selected we cannot exclude that responders differ in experiences by non-responders. In addition, most of the studies exploring the reflecting thinking of medical students have traditionally used written narratives in the form of reflective journals, blogs or assignments. In the present study, critical incidents were assessed through individual interviews and this might have prompted students to narrate the facts more than critically evaluating and reflecting on them.

### Recommendations for practice

In terms of reflection, less than half of the students describing a critical incident reflected upon it. This indicates that students who experience challenging or conflicting situations in a clinical setting do not process them at a cognitive level, which is important in order to achieve academic or personal benefits from the experience. Critical reflection has been linked with better professional development (Andersen, Hansen, Søndergaard, & Bro, 2008; Boenink, Oderwald, De Jonge, Van Tilburg, & Smal, 2004; Mann, Gordon, & MacLeod, 2009; Plack, Driscoll, Marquez, & Greenberg, 2010). If medical students do not engage in reflection to the level of integrating the learning from the reflection into future practice, then it is likely they are not progressing to the reconstructive phase of the clinical learning, where behavior change occurs. Through reflection medical students can gain new insights regarding clinical practice through self-awareness and critical, reflective evaluation (Carr & Carmody, 2006). As a result, many educational institutions have incorporated the ability to reflect as an objective of their curricula, premised on a belief that reflective thinking is something that can be developed rather than a stable personality trait (Sandars, 2009). In addition, the fact that reflection at different levels can be assessed means that it can be potentially improved (Boenink et al., 2004).

## Conclusions

Medical students are commonly exposed to conflicting experiences that can have a profound impact on their professional development. These incidents elicit dilemmas regarding the appropriate course of action and reaction. Even though such incidents provide opportunities for medical students to reflect on professionalism, our research suggests that they are not maximizing such learning experiences. Reflection on experience is an increasingly critical part of professional development and lifelong learning. Teaching students to engage in structured reflection can have diverse practical results; such as better history talking and more effective communication.
